# The mediational role of distracting stimuli in emotional word recognition

**DOI:** 10.1186/s41155-017-0082-8

**Published:** 2018-01-25

**Authors:** C. Moret-Tatay, A. Lami, C. R. Oliveira, M. J. Beneyto-Arrojo

**Affiliations:** 1 Universidad Católica de Valencia San Vicente Mártir, calle Guillem de Castro 175, 46008 Valencia, Spain; 2 Universidad de València, Avenida Blasco Ibáñez, 46021 Valencia, Spain; 3 IMED Passo Fundo, Rua Senador Pinheiro, 304-Vila Rodrigues, Passo Fundo - RS, 99070-220 Brazil

**Keywords:** Word recognition, Emotional valence, Mediation, Response times

## Abstract

Emotions are considered distractions that often prompt subsequent actions. In this way, the aim of this work was to examine the role of distracting stimuli on the relationship of RT and accuracy. In order to do that, a word recognition task was carried out in which emotional valence was manipulated. More precisely, a mediational model, testing how changes in distracting stimuli mediate RT predicting accuracy across emotional conditions, was carried out. The results suggest that changes in task demands should distract from the secondary task to the extent that these task demands implicate and affect accuracy. Moreover, the distracting task seems to mediate between accuracy and the target task under emotional stimuli, showing the negative distracting condition to be the most remarkable effect. Furthermore, neutral distracting latencies did not affect accuracy. Understanding the mechanisms by which emotion impairs cognitive functions has important implications in several fields, such as affective disorders. However, the effects of emotion on goal-directed cognitive processing remain unclear.

## Background

How emotion affects memory is a subject of interest for several areas such as clinical or even the forensic fields. In this way, the effect of emotional valence on word recognition has been described in both theoretical and empirical research. This is an effect that has been reported as a robust one on several aspects of cognition, as well as on behavior (Van Tol, Demenescu, Van der Wee, Kortekaas, Marjan, et al. 2012). Several studies claimed that emotional words might capture more attention than neutral ones (Bowen, Kark, & Kensinger 2017; Sereno, Scott, Yao, Thaden, & O’Donnell 2015). More precisely, and focusing on the particular effect of negative emotional content on word recognition, it has been suggested that negative stimuli elicit slower latencies under this condition. According to Moret-Tatay, Moreno-Cid, Argimon, Quarti Irigaray, Szczerbinski, et al. (2014), this is a plausible result suggesting that an automatic vigilance process might operate to engage attention longer. In particular, the literature (León Gordillo, Martínez, Hernández, Cruz, Meilán, et al. 2010; Meng, Zhang, Liu, Ding, Li, et al. 2017) has suggested that the “normal population” might be predisposed to direct their attention to negative stimuli, supporting the idea that the negative emotional charge could have an essential role in our evolution.

Another remarkable issue regarding this topic is that emotional words seem to be remembered better than neutral ones (Ferré, García, Fraga, Sánchez-Casas, & Molero 2010; Herbert & Kissler, 2010). Not surprisingly, it has been hypothesized that this rise of arousal might not only have an effect on response time (RT), slowing it down, but also in improving encoding (Ferré, Fraga, Comesaña, & Sánchez-Casas 2015). In other words, to deal with emotional valence could be also considered a cognitive cost. Other authors have stipulated that accuracy and speed processing might be accommodated through several parameters such as decision components and variability (Mueller & Kuchinke 2016; Ratcliff, Smith, Brown, & McKoon 2016). In terms of memory processing, recognition might include some retrieval-based processing (Racsmány, Szőllősi, & Bencze 2017). Specifically, RT has been negatively associated with accuracy (Robinson & Johnson 1996).

As is popularly known, states such as depression and posttraumatic stress disorder are often characterized by increased susceptibility to emotional distraction. It involves conscious and unconscious processes that draw attention away from a task. Moreover, Foerde, Knowlton, and Poldrack (2006) found evidence that memory performance could be modulated by distraction by using functional MRI (fMRI). On a theoretical level, distraction has been described as an important key in theories of emotion regulation (Zhang, Gross, & Hayne 2017).

According to the literature (Dolcos & McCarthy 2006; Schwager & Rothermund 2013), the neural systems try to mediate the detrimental effects of emotional distractors. These might be defined as emotional information that impairs cognitive functions due to a high attentional cost or a detrimental effect on inhibition. In this way, several authors have proven that emotional irrelevant information can capture attention from the task in question, in terms of latency components (Gupta, Hur, & Lavie 2016; Hodsoll, Viding, & Lavie 2011). Although many efforts have been made, it remains unclear whether there would be an effect of emotional valence on accuracy for the irrelevant distractors in conditions of low load. That is to say how emotional valence can interfere with the efficiency of the underlying basic cognitive process related to attention. For this reason, a mediation model among the variables described before was chosen. In this way, the type of analysis is related to a path analysis, which is of interest to examine the relationships between variables. In particular, this is a useful tool for the testing of a model and both direct and indirect effects on a given result (such as mediation and moderation among other relationships), under the basis of multiple regression. Moreover, it can popularly be understood as a particular case of structural equation modeling (SEM). For the purpose of the present study, this method is of interest, as it might allow us to examine the mediational role of distracting stimuli in latencies to emotional stimuli. Due to the above considerations, several results were expected. First of all, we expected a stronger correlation between accuracy and reaction time under emotional conditions, indicating a cognitive cost. Finally, we expected that latencies under distracting stimuli would mediate the recognition in terms of target latencies and accuracy variables, depicting working memory (WM) and inhibition processes.

## Method

### Participants

A sample of 95 university students took part in the experiment (62 women and 33 men, with an average age of 25.12 years and SD = 3.10). In terms of inclusion criteria, all the participants had normal or corrected to normal vision, were native Spanish speakers and did not report cognitive or neurological disorders after a personal interview.

### Materials

The stimuli employed were a selection of words from the *Busca Palabras* database (Davis & Perea 2005). A total of 90 words were divided into three sets of 30 stimuli, based on their scores on emotional valence (positive, negative, or neutral); see Table 1. Employing the same stimuli as Moreno-Cid, Moret-Tatay, Irigaray, Argimon, Murphy, et al. (2015), where stimuli rated 4 or lower were considered to be negative, rated 4 to 6 to be neutral, and rated above 6 to be positive (consistent with previous literature, see Moret-Tatay et al. 2014). From the 90 words selected, 45 words were designated as target (and appeared in the first and second block) and the other 45 as distractors (and appeared only in the second block).

**Table 1:** Average valence for the selected words in the different sets (standard deviation in parenthesis)

Valence/condition	Neutral	Negative	Positive
Target	4.77 (0.20)	2.86 (0.62)	6.62 (0.53)
Distracting	4.89 (0.40)	2.84 (0.78)	7.2 (0.62)

### Procedure

Participants were tested in a quiet room, in groups of three or four. The presentation of stimuli and recording of response times were controlled by a Windows operating system through the DMDX software (Forster & Forster 2003). The experiment consisted of two phases. In the first phase, 45 target stimuli were randomly presented (divided into 15 stimuli for each of the three valence categories) with short exposures of 2 s each. In the second phase (15 min after the participants were distracted by performing Stroop tasks), 45 target stimuli plus the 45 distracting stimuli were randomly presented. Each word was presented until the participant gave a response or 2000 ms had passed. The participants were instructed to press a button (labeled “Yes”) if the stimulus was a target stimulus and press another button (labeled “No”) if the stimulus was a distractor stimulus. The participants were also instructed to respond as quickly as possible while maintaining a reasonable level of accuracy. The session lasted approximately 30 min.

### Design and data analysis

**Fig. 1:**
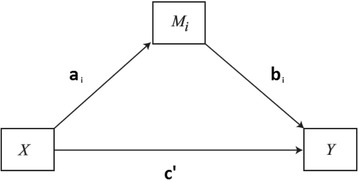
Mediational model under study to test and its paths

A repeated measure design was employed where a classical analysis of variance (ANOVA) explored the impact of stimulus identity (target or distractor) and emotional valence on response latency and accuracy. The statistical analysis was performed using SPSS 20. We conducted a mediational analysis using process macro for SPSS (Hayes 2015) to test the hypothesis that latency changes in distracting stimuli mediate the effect of RT in predicting accuracy. In this way, regression-based mediation procedures were executed employing bootstrapping procedures (MacKinnon & Fairchild 2009; Hayes 2009). This method allows the measuring of the indirect effect that represents the impact of the mediator variable on the stipulated relation by a method of bootstrapping (10000) with confidence intervals. More precisely, a regression coefficient (and associated *t* test) was first calculated on the mediational *M* variable (and its inherent a and b paths), the *X* independent variable on the dependent variable without the inclusion of moderator (c’ path) and the *X* independent variable on the dependent variable after the mediator was included (c path). Figure 1 depicts this analysis in terms of variables and paths.

## Results

The RTs were longer for negative stimuli than positive and neutral ones. Table 2 presents the average reaction times (ms), error rates, and standard deviation for each group of words. In the ANOVA for latency analyses, RTs less than 250 ms and over 1800 ms were excluded (less than 2% of the data set). The 1800 ms cutoff point was adopted for consistency with earlier studies in the field (Moret-Tatay et al. 2014; Moret-Tatay, Leth-Steensen, Irigaray, Argimon, Gamermann, et al. 2016; Moret-Tatay et al. 2016; Perea, Panadero, Moret-Tatay, & Gómez 2012). A 2 × 3 ANOVA was conducted.

**Table 2:** Response time averages (ms), error rate, and standard deviation (SD) for different experimental conditions

Images	Neutral	Negative	Positive
Target	853.79	864.13	843.18
*SD*	140.64	147.50	133.41
*Accuracy*	61.9%	67.2%	69.5%
Distracting	936.77	953.17	938.52
*SD*	170.35	164.52	173.28
*Accuracy*	72.6%	68.3%	74.2%

The ANOVA of RT showed that differences in emotional valence (where negative stimuli was recognized slower) were close to but did not reach statistical significant: *F*_(2, 188)_ = 2.94; MSE = 16,329.33; *η*^2^ = 0.03; *p* = 0.055. Bonferroni’s pairwise comparison approached but did not reached the statistically significant for negative versus positive stimuli (*p* = 0.06). In the case of distracting and target conditions, target stimuli were processed faster than distractors, and this difference reached statistical significance: *F*_(1,94)_ = 61.25; MSE = 1,131,718.40; *η*^2^ = 0.39; *p <* 0.01. Any interaction or error differences did not reach statistical significance (*F <* 1).

On the other hand, the ANOVA of on accuracy showed that differences in emotional valence (where positive stimuli was recognized slower) reached the statistical significant, *F*_(2, 188)_ = 8.33; MSE = 0.015; *η*^2^ = 0.081; *p* < 0.01. In the case of distracting and target conditions, target stimuli were processed faster than distractors, and this difference reached the statistical significance: *F*_(1,94)_ = 5.38; MSE = 0.434; *η*^2^ = 0.054; *p <* 0.05. All Bonferroni’s pairwise comparison between negative versus positive/neutral stimuli were statistically significant for emotion (*p* < 0.05), but neutral versus positive differences did not reach the statistical level. An interaction (emotion*presentation) was found across variables: *F*_(2, 188)_ = 6.99; MSE = 0.016; *η*^2^ = 0.069; *p* < 0.01.

**Fig. 2:**
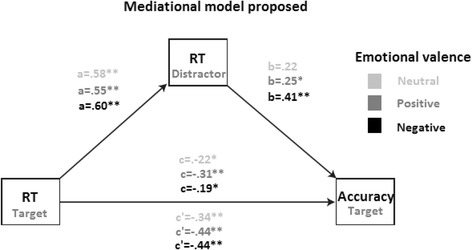
Mediational model testing how changes in distracting stimuli mediate RT predicting accuracy across emotional conditions

Finally, a mediational model was tested. Previously, any relationship between variables was examined through Pearson correlation coefficient (Fig. 2). As depicted in Table 3, changes in latencies to distracting stimuli significantly mediated errors on target stimuli for the negative condition (*F*_(1,93)_ = 53.07; MSE = 0.6435; *R*^2^ = .36; *p <* 0.001), the neutral (*F*_(1,93)_ = 47.83; MSE = 0.6675; *R*^2^ = .34; *p <* 0.001) and the positive one (*F*_(1,93)_ = 40.85; MSE = 0.7022; *R*^2^ = .31; *p <* 0.001). Figure 1 depicts each of the paths for each model. All of them reached the statistical significance, except for the b path in the neutral condition. Any other combination across emotional condition did not reach the statistical significance.

**Table 3:** Pearson coefficients across RT, accuracy, emotional valence, and condition (target and distracting)

			RT						Accuracy					
			Target			Distracting			Target			Distracting		
			Negative	Neutral	Positive	Negative	Neutral	Positive	Negative	Neutral	Positive	Negative	Neutral	Positive
RT	Target	Negative	1	.715^**^	.726^**^	.603^**^	.643^**^	.577^**^	− .197	− .242^*^	− .216^*^	.096	.201	− .033
		Neutral		1	.722^**^	.613^**^	.583^**^	.618^**^	− .076	− .220^*^	− .157	− .034	.109	− .194
		Positive			1	.555^**^	.576^**^	.552^**^	− .209^*^	− .290^**^	− .308^**^	− .043	.028	− .115
	Distracting	Negative				1	.766**	.814**	.145	− .056	.091	− .146	− .102	− .351**
		Neutral					1	.750**	.170	.016	.056	− .118	− .137	− .305**
		Positive						1	.199	− .048	.006	− .227*	− .180	− .416**
Accuracy	Target	Negative							1	.422^**^	.505^**^	− .218^*^	− .171	− .114
		Neutral								1	.490^**^	− .124	− .031	.034
		Positive									1	.080	− .100	− .016
	Distracting	Negative										1	.651^**^	.625^**^
		Neutral											1	.654^**^
		Positive												1

## Conclusions and discussion

Emotions are considered distractions that often prompt subsequent actions. The aim of this work was to examine the role of distracting stimuli on the relationship of RT and accuracy. Understanding the mechanisms by which emotion impairs cognitive functions has important implications in several fields, such as affective disorders. Moreover, and according to Dolcos and McCarthy (2006), the neural systems that mediate the effects of emotion on goal-directed cognitive processing remain unclear.

The results could be described as follows: (i) First of all, changes in task demands should distract from the secondary task to the extent that these task demands implicate and affect accuracy, (ii) the distracting task mediates between the target task and accuracy under emotional stimuli, (iii) negative distracting stimuli seem to have a higher mediational effect than neutral or positive distracting stimuli, and (iv) positive distracting stimuli seem to have a higher mediational effect than neutral distracting stimuli.

As expected from the literature, latencies to distracting words presented higher RT. This indicates a cognitive cost in rejecting the second demand in a task. Moreover, and as it was hypothesized, RTs were inversely correlated to accuracy in most of the cases, in particular, under emotional conditions (as expected from previous literature (León Gordillo et al. 2010; Moreno-Cid et al. 2015). In this way, a mediational effect was found from distracting stimuli, except for the neutral ones. This might emphasize the role of emotion on memory, as a bias described before. Moreover, this type of approach could be considered as an extra value of the present work, as traditional analyses of variance, such as ANOVA, might be implemented by taking advantage of other more sophisticated analyses (Moret-Tatay et al. 2017).

In sum, our results showed that distraction should be intermediate under emotional conditions. Moreover, a stronger prediction from the negative stimuli was found. This hypothesis has received strong empirical support that negative stimuli generally hold more attention than neutral or positive stimuli, even for mood-congruent cognitions (Van Dillen & Koole 2007). This work also presents potential limitations. There are remarkably high error rates, which indicate that the task might be difficult for participants indicating a floor effect. For future research, it would be interesting to develop an easier series of experiments that might also examine the role attentional demands as indicated by several authors (Moret-Tatay et al. 2014; Moret-Tatay & Perea 2011; Navarro-Pardo, Navarro-Prados, Gamermann, & Moret-Tatay 2013) and its possible interactions with emotional valence.
